# Incorporation of a Horizontally Transferred Gene into an Operon during Cnidarian Evolution

**DOI:** 10.1371/journal.pone.0031643

**Published:** 2012-02-06

**Authors:** Catherine E. Dana, Kristine M. Glauber, Titus A. Chan, Diane M. Bridge, Robert E. Steele

**Affiliations:** 1 Department of Biological Chemistry, University of California Irvine, Irvine, California, United States of America; 2 Developmental Biology Center, University of California Irvine, Irvine, California, United States of America; 3 Department of Biology, Elizabethtown College, Elizabethtown, Pennsylvania, United States of America; American University in Cairo, Egypt

## Abstract

Genome sequencing has revealed examples of horizontally transferred genes, but we still know little about how such genes are incorporated into their host genomes. We have previously reported the identification of a gene (*flp*) that appears to have entered the *Hydra* genome through horizontal transfer. Here we provide additional evidence in support of our original hypothesis that the transfer was from a unicellular organism, and we show that the transfer occurred in an ancestor of two medusozoan cnidarian species. In addition we show that the gene is part of a bicistronic operon in the *Hydra* genome. These findings identify a new animal phylum in which trans-spliced leader addition has led to the formation of operons, and define the requirements for evolution of an operon in *Hydra*. The identification of operons in *Hydra* also provides a tool that can be exploited in the construction of transgenic *Hydra* strains.

## Introduction

Horizontal transfer of genes is widely accepted as a significant feature of genome evolution in prokaryotes [1], and likely in unicellular eukaryotes as well [2]. However, it has been much more difficult to build convincing cases for horizontal gene transfer (HGT) into animal genomes. The amount of HGT in animals is expected to be much less than in unicellular organisms. This expectation is due to the absence in animals of the facile routes for DNA uptake seen in prokaryotes and the necessity of targeting the germ line, which is segregated in most metazoans, in order for a horizontally transferred gene to be propagated [3].

The most commonly used evidence for HGT is anomalous phylogenetic distribution of the gene being considered. Whether such a distribution constitutes strong evidence for HGT depends on the number of genomes being assessed. A relatively small number of animal genomes have been sequenced, making models invoking gene loss [4] viable contenders for explaining anomalous phylogenetic distributions in animals. Ultimately one makes the argument for HGT based on parsimony – with a single horizontal transfer event being considered more parsimonious than multiple secondary losses of a gene. Without information on how difficult it is for a gene to be lost versus the difficulty of being horizontally transferred into a given animal genome, it is not known whether this assumption is correct.

The genome of the cnidarian *Hydra* presents a potentially fertile hunting ground for identifying horizontal gene transfers into an animal genome. In the adult *Hydra* polyp, all cells are separated from the environment by no more than a single cell layer [5]. Thus all cells are readily exposed to exogenous sources of DNA (e.g. bacteria and unicellular eukaryotes). *Hydra* propagates primarily by asexual budding and its germ line is not segregated [6], [7], [8], features that greatly increase the potential for a horizontally transferred gene spreading within the population. Finally, *Hydra* mRNAs undergo trans-spliced leader addition [9], which gives the animal the potential for having operons [10]. Operons provide an opportunity for a gene entering the genome by a horizontal route to “piggy-back” onto an existing gene and thus to be expressed without need for its own promoter. This potential for immediate incorporation into the genetic circuitry of the animal makes it possible for the gene to come under selection for a function quickly upon entering the genome.

Sequencing of the *Hydra* genome has led to the identification of putative horizontal gene transfers from bacteria [11]. Habetha and Bosch [12] have reported the presence of a peroxidase gene in *Hydra* that may have entered by horizontal gene transfer from a plant. We identified a *Hydra* gene, called *flp*, whose only homologues at the time of its discovery were in the genome of the parabasilid protist *Trichomonas vaginalis* [13]. The function of the *flp* gene is unknown, although its expression has been shown to respond to iron levels in *T. vaginalis* [14], and the amino acid composition of the 14.8 kDa *flp* protein suggests that it might be a metal-binding protein [13]. We report here additional findings regarding the *flp* gene that provide insight into its evolutionary history and its incorporation into the *Hydra* genome. In addition, we demonstrate that *Hydra* has operons in its genome, and that the requirements for forming an operon in *Hydra* are surprisingly simple.

## Results

### Phylogenetic distribution of the *flp* gene

**Figure 1:**
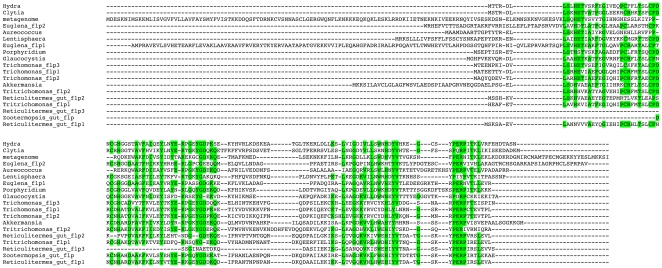
Alignment of *flp* amino acid sequences. Sequences were aligned using MUSCLE [50]. Amino acids highlighted in green are conserved in a majority of the sequences. Sequence sources are as follows: meta, translated from GenBank accession number AACY020544127 (DNA from the Sargasso Sea); Euglena_1, translated from *Euglena gracilis* EST with accession number EC679450; Euglena_2, translated from *Euglena gracilis* EST with accession number EC678321; Glaucocystis, translated from *Glaucocystis nostochinearum* EST with accession number EC122554; Tf_1, translated from *Tritrichomononas foetus* EST with accession number CX156355; Tf_2, translated from *Tritrichomononas foetus* EST with accession number CX157959; Reticulitermes_gut_1, translated from *Reticulitermes flavipes* symbiont ESTs with accession numbers FL643370 and FL643898; Reticulitermes_gut_2, translated from *Reticulitermes flavipes* symbiont ESTs with accession numbers FL637453 and FL638405; Reticulitermes_gut_3, translated from *Reticulitermes flavipes* symbiont EST with accession number GO904605; Tv_1, from *Trichomonas vaginalis* protein XP_001316516; Tv_2, from *Trichomonas vaginalis* protein XP_001322305; Tv_3, from *Trichomonas vaginalis* protein XP_001324076; Clytia, translated from a *Clytia haemispherica* EST with accession number FP933787. Zootermopsis_gut, translated from *Zootermopsis* symbiont EST with accession number EG751663; Aureococcus, from *Aureococcus anophagefferens* protein EGB07434; Lentisphaera, from *Lentisphaera araneosa* protein ZP_01876528; Porphyridium, translated from *Porphyridium purpureum* EST with accessison number HS847715; Akkermansia, from *Akkermansia muciniphila* protein YP_001878433. Neither of the two *Euglena* ESTs or the two *Tritrichomonas foetus* ESTs contained a start codon preceded by an in frame stop codon. Thus it is unclear if the amino terminal sequences shown here are correct for these species. The Reticulitermes_gut_3 and Zootermopsis_gut ESTs encode only a portion of the protein.

**Figure 2:**
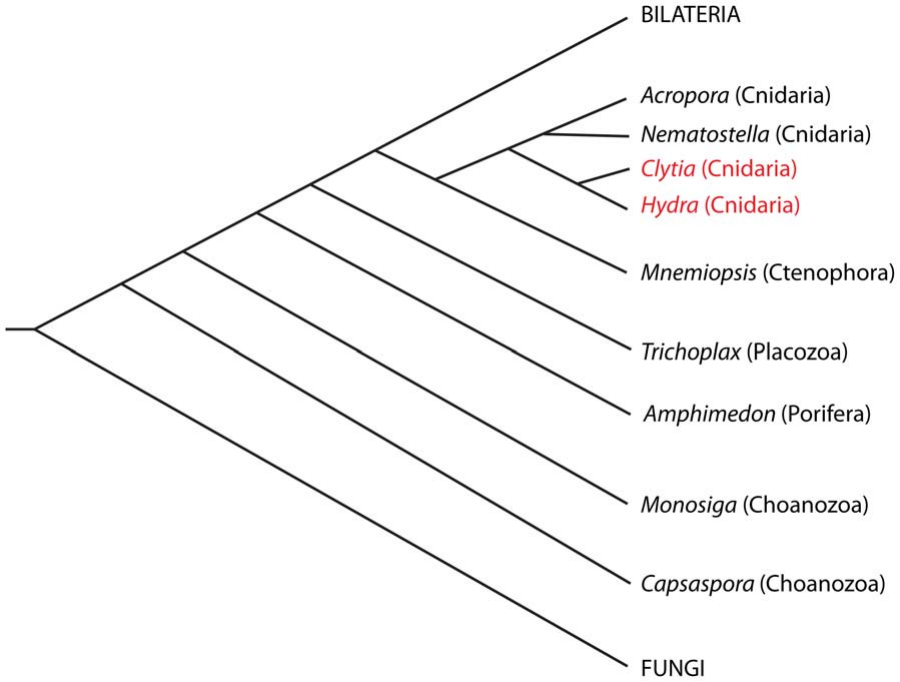
Distribution of the *flp* gene among opisthokonts. Evolutionary relationships of opisthokonts are shown, with the two medusozoans that contain a *flp* gene indicated in red. The placement of Ctenophora among opisthokonts is still unresolved. We have chosen the position proposed by Philippe et al. [55]. The position of *Capsaspora* is as proposed by Shalchian-Tabrizi et al. [56].

Since our original report of a *Hydra* homologue of the *Trichomonas vaginalis flp* genes [13], *in silico* screens of genome sequences and ESTs have revealed *flp* genes in several additional organisms (Fig. 1). These include a marine bacterium (*Lentisphaera araneosa*), a human gut bacterium (*Akkermansia muciniphila*), a glaucophyte (*Glaucocystis nostochinearum*), a euglenid (*Euglena gracilis*), a pelagophyte (*Aureococcus anophagefferens*), a red alga (*Porphyridium purpureum*), an additional parabasilid protist (*Tritrichomonas foetus*), termite gut symbionts, and a metagenome from the Sargasso Sea. The gene is also present in the cnidarian *Clytia hemisphaerica*. Like *Hydra*, *Clytia* is a member of the subclass Hydroidolina in the cnidarian subphylum Medusozoa [15]. The *flp* gene is absent from the genomes of the anthozoan cnidarians *Nematostella vectensis* [16] and *Acropora digitifera* [17]. The divergence of Anthozoa and Medusozoa occurred at the base of the cnidarian radiation [18], [19]. The *flp* gene is also absent from the genomes of the choanoflagellate *Monosiga brevicollis* [20], *Capsaspora owczarzaki* (an opisthokont that diverged between fungi and choanoflagellates) (GenBank Accession Number ACFS00000000), the sponge *Amphimedon queenslandica* [21], the placozoan *Trichoplax adhaerens* [22], the ctenophore *Mnemiopsis leidyi* (Joseph Ryan, personal communication), and all publicly available bilaterian animal, plant, and fungal genome sequence and EST datasets. Fig. 2 shows the evolutionary relationships of the opisthokonts for which we have presence/absence information regarding the *flp* gene.

**Figure 3:**
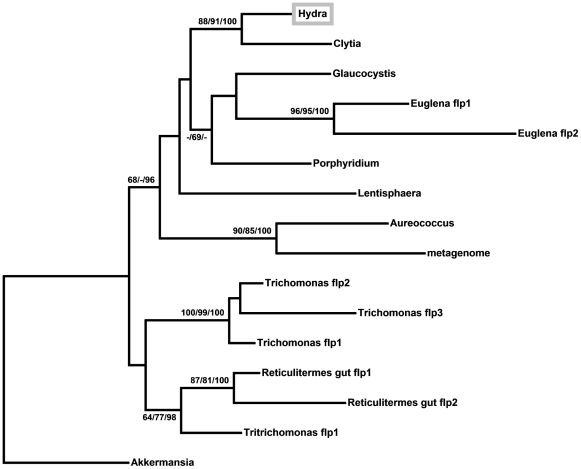
Phylogenetic relationships among *flp* proteins. Relationships are shown as an unrooted maximum likelihood phylogram. The numbers at the nodes indicate maximum likelihood bootstrap support values/maximum parsimony bootstrap support values/Bayesian posterior probabilities. A dash indicates a value less than 60. Nodes without numbers have bootstrap support and Bayesian posterior probabilities of less than 60.

From a phylogenetic analysis of *flp* protein sequences (Fig. 3), we conclude that the three *flp* genes in *Trichomonas vaginalis* are paralogues, as are the two *flp* genes in *Euglena*. This analysis also shows that the *Clytia* and *Hydra flp* genes are vertically descended from a common ancestor within Medusozoa.

### Genomic organization of the *Hydra flp* gene

**Figure 4:**
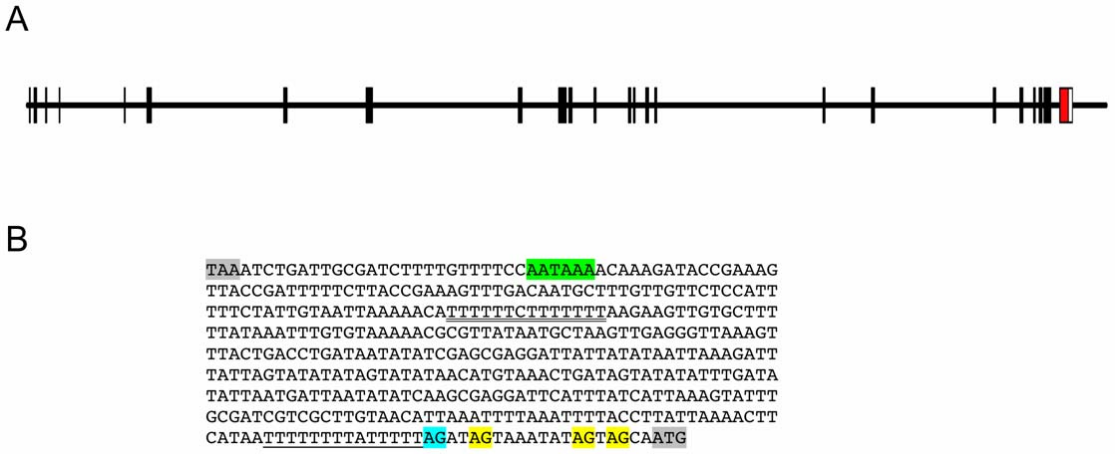
RFC140/*flp* exon-intron organization (A) and intergenic region sequence (B). The RFC140 gene exons are shown in black. The single *flp* exon and 3′ UTR are shown in red and white respectively. The coding sequence of the RFC140 gene has been deposited in GenBank under accession number FJ154842. Annotations in Panel B are as follows: gray, RFC140 stop codon and *flp* start codon; green, putative polyadenylation signal; double-underline, T-rich sequence; single-underline, polypyrimidine tract associated with the trans-splicing acceptor dinucleotide; blue, trans-splicing acceptor dinucleotide; yellow, additional AG dinucleotides changed to AA in mutant plasmid.

The *Hydra flp* gene was originally identified from ESTs [13]. Subsequently we amplified the coding sequence of the *flp* gene from *Hydra* genomic DNA and found that it lacks introns, as do all three copies of the *flp* gene in *Trichomonas*. Using the genome assembly from the *Hydra* genome project [11], we identified the region containing the *flp* gene. Immediately upstream of the *flp* gene is a gene encoding the 140 kDa subunit of replication factor C (RFC140) (Fig. 4A). From mapping of ESTs onto the genome sequence and blasting with RFC140 sequences from other metazoans, we determined the coding sequence (GenBank Accession Number FJ154842) and exon/intron structure of the *Hydra* RFC140 gene. From EST sequences, we found that the RFC140 mRNA, like the *flp* mRNA [13], is trans-spliced. RFC140 ESTs containing spliced leaders SL-B1, SL-B3, SL-C, and SL-F3 [9], [11] were identified. The stop codon of RFC140 is only 417 nucleotides upstream of the trans-splicing acceptor dinucleotide of the *flp* gene (Fig. 4B). This 417 nucleotide sequence must include the RFC140 3′ UTR (between 63 and 83 nucleotides in length if the predicted polyadenylation signal is correct and polyadenylation begins 10–30 nucleotides downstream from this signal). Thus the distance between the end of the RFC140 gene and the beginning of the *flp* gene is less than 417 nucleotides. This short distance and the fact that the *Hydra flp* mRNA undergoes trans-spliced leader addition [13], suggested that the *flp* gene is in an operon with the RFC140 gene. Upstream of the putative trans-splicing acceptor in the *flp* gene is a T-rich (U-rich in RNA) sequence (doubly underlined in Fig. 4B). Huang et al. [23] have shown that a U-rich sequence upstream of a trans-spliced gene is essential for splicing in operons in *C. elegans*. Candidate operons have been reported in other metazoans that undergo trans-spliced leader addition [24], [25], [26], including *Hydra* [11], but none have been experimentally verified. Using multiple approaches, we tested the hypothesis that the *Hydra* RFC140 and *flp* genes are in an operon.

### The RFC140 and *flp* genes are in an operon

To test whether RFC140 and *flp* are in an operon, we constructed a plasmid in which a single promoter is located upstream of two fluorescent protein genes, with these genes separated by the RFC140/*flp* intergenic sequence. In order to construct the plasmid, we needed a fluorescent protein gene in addition to the GFP gene previously used in *Hydra* and contained in the plasmid hoTG [27], [28]. Due to *Hydra*'s strongly A+T-biased codon usage [29], we had a version of the DsRed2 red fluorescent protein gene synthesized using *Hydra* codon and codon pair preferences. The sequence of the gene was generated using computationally optimized DNA assembly (CODA) [30] and commercially synthesized by CODA Genomics (now Verdezyne).

**Figure 5:**
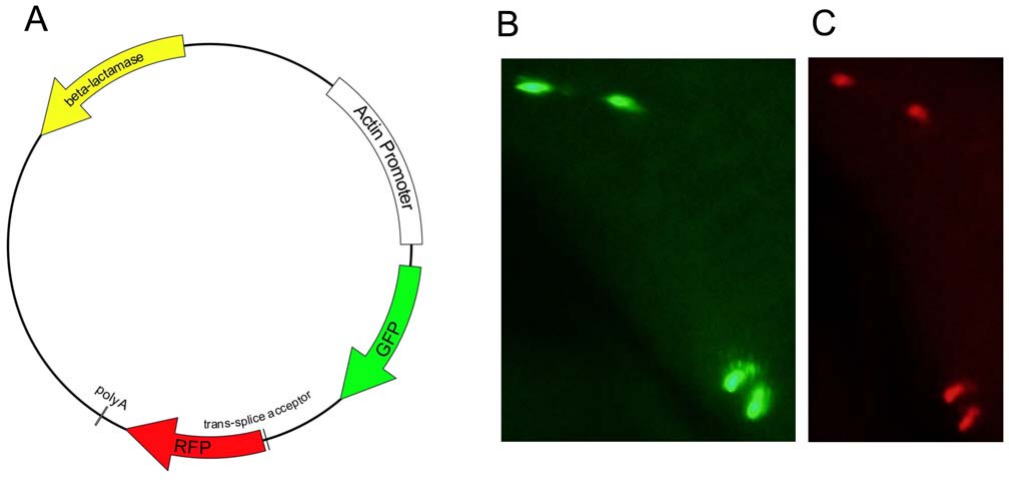
Structure of the operon plasmid and expression of the plasmid following particle bombardment. Panel A shows the structure of pHyVec7, the artificial operon plasmid. Panels B and C show expression of GFP (B) and DsRed2 (C) in four ectodermal epithelial cells in the body column of a live polyp that was bombarded with gold particles coated with the pHyVec7 DNA.

**Figure 6:**
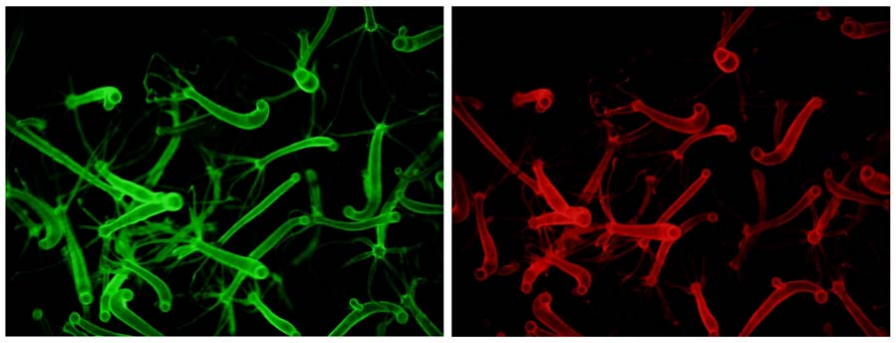
Stably transgenic *Hydra* produced with pHyVec7. Panels show expression of GFP (left) and DsRed2 (right) in a stably transgenic line produced by injecting an embryo with pHyVec7. In this line, only the ectodermal epithelial cell lineage is transgenic.

**Figure 7:**
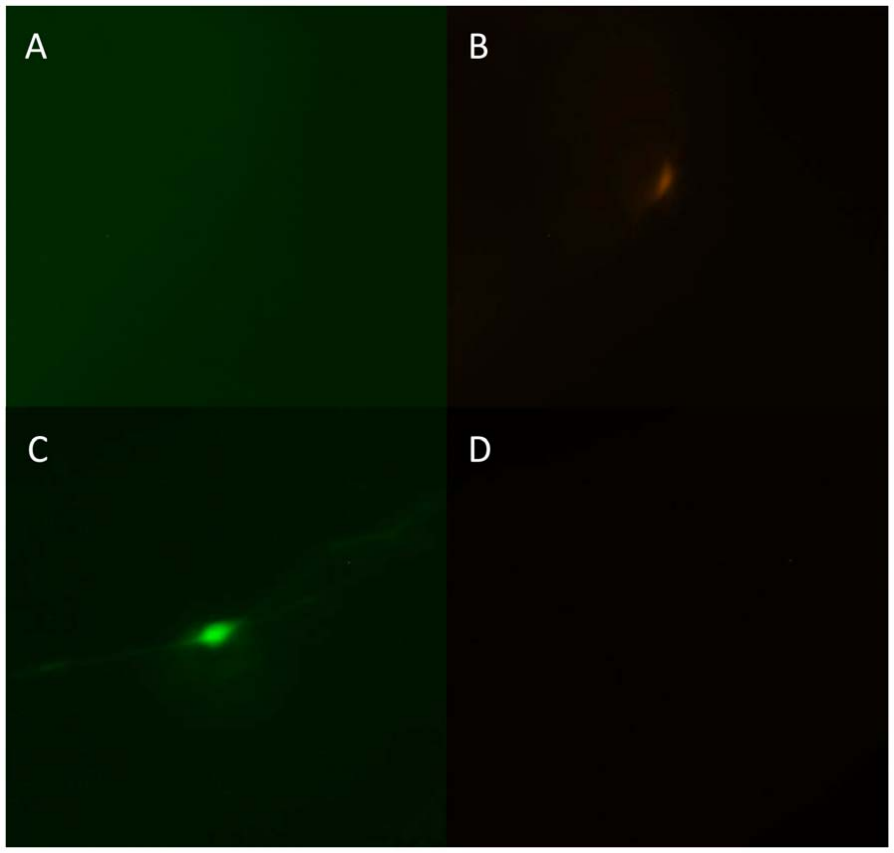
Tests of the intergenic region for promoter activity and of an operon construct with a mutated trans-splicing acceptor dinucleotide. The intergenic region was placed upstream of the green fluorescent protein gene and the resulting plasmid was introduced into adult *Hydra* polyps by particle bombardment together with a plasmid in which DsRed2 expression is driven by an actin promoter. Panels A and B show an ectodermal epithelial cell in the body column of a bombarded polyp that expresses DsRed2 from the control plasmid (B) but not green fluorescent protein from the plasmid with the intergenic region (A). Panels C and D show an ectodermal epithelial cell in the body column of a polyp that was bombarded with the operon construct in which the trans-splicing acceptor dinucleotide was mutated. The cell expresses the upstream green fluorescent protein gene (C) but not the downstream DsRed2 gene (D).

Using the GFP gene from hoTG, the promoter, 3′ UTR, and polyadenylation site from a *Hydra* cytoplasmic actin gene [29], the synthetic DsRed2 gene, and the intergenic region between the RFC140 and *flp* genes, we constructed an operon plasmid with the GFP gene in the upstream position and the DsRed2 gene in the downstream position (Fig. 5A). The resulting plasmid (pHyVec7, GenBank accession number EF539830) was introduced into *Hydra* using particle bombardment [27]. Cells in the bombarded animals that expressed GFP were always positive for DsRed2 expression (Fig. 5B, C). To further confirm our finding of co-expression, we generated a stably transgenic line with transgene expression in the ectoderm. This line co-expresses GFP and DsRed2 (Fig. 6). To rule out the possibility that a promoter is present in the RFC140/*flp* intergenic region and is responsible for expression of *flp*, we made a plasmid in which the intergenic region is located upstream of the GFP gene. *Hydra* bombarded with this plasmid, mixed with a separate plasmid expressing DsRed2 under control of the actin promoter, contained cells that were red but not green (Fig. 7A, B). Thus the intergenic region does not contain a promoter.

**Figure 8:**
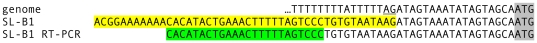
RNA from the pHyVec7 transgene is trans-spliced. RNA from the operon transgenic line was reverse-transcribed to produce first strand cDNA. The cDNA was amplified with primers for the spliced leader SL-B1 and the DsRed2 coding sequence. The top line shows sequence between the RFC140 and *flp* genes extending from the polypyrimidine tract associated with the trans-splicing AG acceptor dinucleotide (doubly underlined) to the start codon of the *flp* gene (shaded in gray). The second line shows the sequence from a previously identified *flp* EST containing the SL-B1 spliced leader sequence [13]; the spliced leader sequence is highlighted in yellow, and the *flp* start codon is shaded. The third line shows sequence from the RT-PCR product obtained from the pHyVec7 transgenic line using SL-B1 and DsRed2 primers. The sequence corresponding to the SL-B1 primer is highlighted in green, and the DsRed2 start codon is shaded.

To confirm that trans-spliced leader addition to the downstream transcript was occurring in this artificial operon, we carried out RT-PCR on RNA from the transgenic line with a primer for one of the spliced leaders (SL-B1) known to be used on the *flp* RNA [9] and a primer for the DsRed2 coding sequence. The sequence of the resulting PCR product confirmed that the DsRed2 mRNA was trans-spliced at the same site as *flp* mRNA (Fig. 8).

While trans-splicing of a bicistronic mRNA to produce two mRNAs is the most straightforward explanation for co-expression of the GFP and DsRed2 genes, alternative explanations include internal ribosome entry or translational read-through of a bicistronic mRNA [31]. The later explanation seems unlikely as there are nine in-frame stop codons between the GFP stop codon and the DsRed2 start codon. If the bulk of DsRed2 expression is due to internal ribosome entry, prevention of trans-splicing should not affect DsRed2 expression. To test whether expression of DsRed2 is dependent on trans-splicing, we mutated the AG dinucleotide that is the acceptor for trans-splicing (see Fig. 3B) to AA. We also mutated the three AG dinucleotides between the trans-splicing acceptor and the DsRed2 start codon to AA in order to prevent trans-splicing from occurring at alternative sites. Particle bombardment of *Hydra* with the mutated construct yielded cells expressing GFP but not DsRed2 (Fig. 7C, D). Thus blocking of trans-splicing prevents expression of the DsRed2 gene but has no effect on GFP expression. From this result, we conclude that *flp* gene expression requires trans-splicing and that expression of the RFC140 gene does not require trans-splicing of *flp*.

## Discussion

How frequently horizontal transfer of genes into the genomes of metazoans occurs, what taxa the genes come from, and the routes they take to get into the target genome are questions about which we have very little information. All sequenced metazoan genomes contain some genes that show anomalous phylogenetic distributions, consistent with horizontal transfer. Absence of introns in such genes is taken as additional evidence that they were horizontally transferred, either from a prokaryote or by retrotransposition. These properties, while expected of a horizontally transferred gene, cannot be taken as proof of such transfer. Secondary loss is always a possibility, and is a particularly viable hypothesis when one is examining the genome of an organism from a part of the metazoan tree that is not well-sampled [4]. The strongest case for horizontal transfer of a gene into a metazoan would be if: (1) the gene has an anomalous phylogenetic distribution; (2) the gene lacks introns; (3) the gene has no homologues in a substantial number of taxa that diverged immediately before and after the organism being considered; (4) there is a reasonable hypothesis for how the gene entered the germ line. The final criterion is the most difficult to satisfy for most metazoans, since the germ line is usually segregated early during embryonic development and a route for an exogenous gene to reach it is not easily imagined.

The *Hydra flp* gene provides a particularly compelling case for horizontal gene transfer in a metazoan. The gene has not been identified by genome or EST sequencing in any opisthokonts other than *Hydra* and *Clytia*. This includes genome sequences from metazoan phyla that diverged immediately before and after Cnidaria. The *Hydra flp* gene lacks introns, a feature that is consistent with either retrotransposition or acquisition from an organism that lacks introns. The known bacterial *flp* genes and the *flp* genes in *Trichomonas vaginalis* lack introns. In *Hydra*, the germ line remains unsegregated throughout the life of the animal. The germ cells in the adult polyp arise from the interstitial cell lineage, which contains multipotent stem cells that give rise to some classes of somatic cells (e.g. nerve cells and nematocytes) in addition to germ cells [32]. These stem cells arise during embryogenesis [33], divide continuously in the adult, and are transmitted to the progeny produced asexually by budding. An adult *Hydra* polyp contains about 3000 such multipotent stem cells [34], which have a cell cycle of about 24 hours [35]. A horizontally transferred gene that is incorporated into a single such multipotent stem cell could be present both in gametes and in the stem cells of asexually-produced progeny. An interstitial cell lineage similar to that seen in *Hydra* is present in at least some other hydrozoans but not in other cnidarian classes [36]. *Clytia* and *Hydra* share this feature [37], and thus we assume that their last common ancestor had this feature as well. Phagocytosis by multipotent stem cells in *Hydra* has not been reported, but the interstitial cell that becomes the oocyte is phagocytic [38], [39], and the phagocytic ability of various cell types in the medusozoan ancestor of *Hydra* and *Clytia* is obviously not known. Bacteria have been found between and within cells in *Hydra* [40], [41], [42]

The scenario we envision is that a *flp* gene entered a medusozoan genome from the genome of a unicellular organism ingested or associated with the host and that it entered a cell in the lineage that gives rise to the germ line (i.e. the interstitial cell lineage). We cannot, however, rule out the possibility that the *flp* gene was acquired from a unicellular organism by a virus, which then carried it into a cnidarian host. Virus-like particles have been reported in *Hydra* [43]. The simplest version of events has the gene landing immediately downstream of the RFC140 gene. This would allow immediate expression of the gene under control of the RFC140 gene promoter. This scenario requires that the host carried out trans-splicing, which the ancestor of *Clytia* and *Hydra* did [11], [44]. Support for this scenario will be provided if the *flp* gene is in an operon with the RFC140 gene in other medusozoans.

The unicellular organisms in which a *flp* gene has been identified are not taxonomically close to each other (except for *T. vaginalis* and *T. foetus*) and the phylogenetic tree for *flp* (Fig. 3) does not provide information on which clade of unicellular organisms was the likely source of the *flp* gene in cnidarians. It is possible that the *flp* gene has moved horizontally among unicellular organisms as well, complicating the understanding of its evolutionary history.

While our data are consistent with the *flp* gene entering the cnidarian lineage after the divergence of Anthozoa and Medusozoa, a caveat associated with this conclusion is that only two anthozoan genome sequences have been published [16], [17]. It is possible that the *flp* gene was present in the stem cnidarian and that the anthozoans whose genomes have been sequenced have secondarily lost the gene. As more cnidarian genome sequences become available, it should be possible to determine with some precision when the gene entered the cnidarian lineage and whether any secondary loss has occurred.

Our results with an artificial operon in *Hydra* are in contrast to findings in the nematode *Brugia malayi*, in which synthetic operons have also been tested. In *Brugia*, a synthetic operon was constructed in which the upstream gene encoded firefly luciferase and the downstream gene encoded *Renilla* luciferase [45]. Both genes were expressed, but surprisingly the downstream gene was not trans-spliced. This appears to be due to the requirement for a sequence motif in an intron of the downstream gene for proper trans-splicing [46], [47]. Our results show that trans-splicing of the downstream gene in the *Hydra* RFC-*flp* operon does not depend on any sequences in or 3′ to the *flp* gene, since the *flp* gene is absent and the 3′ UTR in the transgene construct is from a *Hydra* cytoplasmic actin gene. By obtaining successful trans-splicing of the bicistronic RNA produced by our construct, we have demonstrated that everything necessary to produce a functional operon is contained in the intergenic region. This result suggests that evolution of a bicistronic operon in *Hydra* is a relatively simple process, not requiring introns in either of the two genes, nor trans-splicing of the upstream gene (the cytoplasmic actin gene that was the source of the promoter in our operon construct does not undergo trans-splicing). Additional facilitation is provided by the A+T-richness of the *Hydra* genome, which should aid in the generation of a polypyrimdine tract (using T) near the splice acceptor dinucleotide and a T-rich sequence in the intergenic region which may be required for trans-splicing.

The ability to construct artificial operons in *Hydra* has practical applications. Creation of transgenic *Hydra* expressing genes whose proteins cannot be fused to a fluorescent protein is problematic. Transgenic animals are initially mosaic [28]. Without a fluorescent marker, it is impossible to identify transgenic tissue and thus to track the formation of fully transgenic animals by asexual propagation. The presence of operons in *Hydra* offers a solution to this problem. By placing the gene of interest upstream of a fluorescent protein gene in a bicistronic operon, expression of the fluorescent protein gene serves as a proxy for expression of the gene of interest. To allow easy cloning of genes into the upstream position of the operon, the plasmid vector pHyVec11 was constructed (GenBank accession number EU183365). In pHyVec11 a linker replaces the GFP gene in pHyVec7. The linker contains an NheI site, a HpaI site, and a BamHI site. This allows insertion of genes engineered with an NheI site upstream of the start codon and a blunt end (compatible with HpaI) or a BamHI-compatible site after the stop codon. The resulting construct will express the gene of interest in unfused form and DsRed2. Thus transgenic animals that express DsRed2 will also express the gene of interest. We have used pHyVec11 to express several genes in transgenic *Hydra* (Steele et al., unpublished observations), indicating that it functions as predicted.

## Materials and Methods

### *Hydra* strains and culture

*Hydra magnipapillata* strain 105 and *Hydra vulgaris* strain AEP were cultured using standard methods, including feeding with *Artemia* nauplii [48]. *H. magnipapillata* strain 105 is the strain that was used for genome sequencing [11]. *H. vulgaris* AEP is the strain used for making transgenic *Hydra* [28]. The phylogenetic relationship between these two strains, both members of the Vulgaris clade, is described in Martínez et al. [49].

### Synthetic DsRed2 gene

A DsRed2 gene with *Hydra* codon and codon pair preferences was synthesized commercially by CODA Genomics (now Verdezyne) using the method of Larsen et al. [30] and a dataset of codons and codon pairs generated from several hundred *Hydra* genes. The sequence of the gene has been deposited in GenBank under accession number EF451141.

### Expression Plasmid Constructions

Expression plasmids used in this study were originally derived from hoTG [27], [28], a plasmid that contains ∼1.5 kb of a *Hydra* actin gene promoter, a green fluorescent protein gene, and the 3′ UTR and polyadenylation site from the same actin gene as the promoter. The pHyVec1 plasmid was constructed by excising the expression cassette from hoTG at NsiI sites in the actin promoter and the actin 3′ flanking sequence, blunting these sites, and cloning the resulting fragment into pBluescript II SK+ blunted at the KpnI and SacI sites of the multiple cloning site. The actin promoter fragment in pHyVec1 has had ∼500 bp deleted from the 5′ end of the promoter present in hoTG. The actin 3′ flanking sequence in pHyVec1 has had ∼400 bp deleted from the 3′ flanking sequence present in hoTG. The pHyVec5 plasmid was constructed by replacing the GFP gene in pHyVec1 with the synthetic DsRed2 gene described above. The pHyVec7 plasmid was constructed using a combination of PCR and standard recombinant DNA methods. When needed, appropriate restriction sites were added to DNA fragments by PCR. The intergenic sequence between the *flp* and RFC140 genes was isolated by amplification from *H. magnipapillata* strain 105 genomic DNA. The intergenic fragment was inserted between the actin promoter and the DsRed2 gene in pHyVec5. The GFP gene was then inserted between the actin promoter and the intergenic segment to yield the completed operon. The sequence of pHyVec7 has been deposited in GenBank under accession number EF539830.

### DNA particle bombardment

Introduction of transgenes into adult *Hydra* polyps by particle bombardment [27] was carried out using a Bio-Rad Biolistic PDS-1000/He Particle Delivery System as follows. Approximately 200 polyps of the 105 strain of *Hydra magnipapillata* that had been starved for 24–48 hours were placed in a 35 mm plastic dish and the culture medium removed. By use of a disposable plastic inoculating loop, the polyps were pushed into a pile in the center of the dish. Gold particles (1 micron; Bio-Rad) were washed as suggested by the supplier and stored at 60 mg/ml in 50% glycerol at −20°C. Coating of gold particles with DNA and preparation of the macrocarriers were carried out immediately before use as described in the Bio-Rad user manual. The Particle Delivery System was evacuated using house vacuum (approximately 25 inches of mercury), and 650 psi rupture disks were used. Each batch of polyps was bombarded five times. Between bombardments, the polyps were gently washed by addition of a small volume of *Hydra* medium to the dish. After washing, the medium was removed and the polyps were again concentrated in the center of the dish using a plastic inoculating loop. Following the last bombardment, the polyps were transferred to a 100 mm Petri dish containing *Hydra* medium and placed at 18°C. On the following day, the polyps were transferred to fresh *Hydra* medium. Two to four days following bombardment, live animals were photographed in *Hydra* medium in a six well tissue culture plate using either an Olympus IX inverted microscope or an Olympus SZX-ILLD2-100 stereomicroscope. Because the gold particle containing the DNA must hit the nucleus in order for the transgene to be expressed [27], many polyps show no expression and those that do have only one to a few expressing cells.

### Generation of Transgenic *Hydra*

Transgenic *Hydra* lines were generated essentially as described by Wittlieb et al. [28]. Polyps that had initiated egg production were collected from mass cultures of *Hydra vulgaris* strain AEP and placed in a Petri dish with polyps of the same strain that had testes. A single blastomere of embryos at the 2–8 cell stage was injected with approximately 0.2 nanoliters of plasmid DNA (0.5 µg/µl) using a Narishige IM-300 Microinjector. Plasmid DNA for injection was prepared using a Qiagen EndoFree Plasmid Giga Kit. The DNA was resuspended in filter-sterilized Milli-Q water and stored at −20°C. Injections were carried out using needles pulled on a Model P-87 Flaming/Brown Micropipette Puller (Sutter Instruments) from filament-containing capillary tubing (Sutter Instruments, catalog number BF100-50-10). Injections were done in *Hydra* medium and injected embryos were kept individually in microtiter plate wells in *Hydra* medium. The plates were initially incubated for two weeks in the dark at 18°C. The plates were then kept at room temperature (18–21°C) on a 17 hour light, 7 hour dark cycle until the embryos hatched. Newly hatched polyps were fed *Artemia* nauplii. Transgenic polyps were cultured until animals were obtained in which all the cells in the lineage that contained the transgene were transgenic. Live transgenic animals were photographed in *Hydra* medium in a six well tissue culture plate using an Olympus SZX-ILLD2-100 stereomicroscope.

### Mutagenesis

Generation of a construct in which the *flp* gene trans-splicing acceptor dinucleotide was mutated was carried out as follows. The intergenic region between the *flp* and RFC140 genes contains a BsaBI cleavage site nine nucleotides downstream from the RFC140 stop codon. A BstAPI cleavage site is present seven nucleotides downstream of the *flp* ATG start codon. A version of the 436 bp segment between these two sites in which the trans-splicing AG acceptor dinucleotide and the three AG dinucleotides immediately downstream of the splice acceptor were replaced by AA (see Fig. 3B) was synthesized commercially by Blue Heron Biotechnologies. The wild-type intergenic segment in pHyVec7 was replaced by the mutated segment to produce the plasmid pHyVec7-mut. The construct was confirmed by sequencing.

### RT-PCR

Total RNA for RT-PCR was isolated from five polyps of the transgenic line using a Quick-RNA MicroPrep kit (Zymo Research). Oligo dT-primed first strand cDNA synthesis was carried out with reagents from the GeneRacer Core Kit (Invitrogen) and reverse transcriptase from the SuperScript III RT Module (Invitrogen). Amplification of spliced leader-containing DsRed2 cDNA was carried out with AccuPrime *Pfx* polymerase (Invitrogen) and the following primers: SL-B1, CACATACTGAAACTTTTTAGTCCC; DsRed2, TGTGGTGATAAAATATCCCACGC. The resulting amplification product was purified using a DNA Clean & Concentrator Kit (Zymo Research) and ligated into the pCR-Blunt plasmid using a Zero Blunt PCR Cloning Kit (Invitrogen). The ligation mixture was used to transform One Shot TOP10 chemically competent *E. coli* cells (Invitrogen), and transformed cells were selected on LB plates containing kanamycin. The recombinant plasmid was sequenced using M13 forward and reverse primers (Eton Bioscience, Inc.).

### Bioinformatics and Phylogenetic Analyses

Sequences related to *flp* were identified using blastp and tblastn to query the protein, EST, whole-genome shotgun read, and environmental samples databases at NCBI. The protein sequence alignment in Fig. 1 was carried out using the CLUSTALW2 server at the EMBL European Bioinformatics Institute (http://www.ebi.ac.uk/Tools/msa/clustalw2/).

For phylogenetic analyses, amino acid sequences were aligned using Multiple Sequence Alignment by Log-Expectation (MUSCLE) [50] on the server at the EMBL European Bioinformatics Institute (http://www.ebi.ac.uk/Tools/msa/muscle/). The portion of the alignment used for analyses began at the start of the *Hydra* sequence and ended at the last point with sequence present for all proteins. *Reticulitermes* gut 3, *Zootermopsis* gut, and *Tritrichomonas foetus* flp2 sequences were not used in analyses, because they are incomplete at the amino terminal ends.

ProtTest [51], on the server at the University of Vigo (http://darwin.uvigo.es/software/prottest_server.html), was used to select among models of protein evolution. The best fitting model of those evaluated, based on AICc and BIC frameworks, was WAG+G. This model was used for the maximum likelihood and Bayesian analyses. The maximum likelihood analysis was performed using the ATGC Bioinformatics Platform PhyML 3.0 server [52] (http://www.atgc-montpellier.fr/phyml/). Bootstrap values were based on 1000 replicates. Bayesian analysis was performed using MrBayes 3.1.2 [53], with four Markov Chain Monte Carlo chains run for 2,000,000 generations and sampled every 100 generations, and with the first 500,000 generations discarded as burn-in. Parsimony analysis was conducted using PAUP* 4.10 [54], with 1000 bootstrap replicates, each involving a heuristic search with 10 random addition replicates and TBR branch swapping. Amino acid substitutions were weighted using the PAUP protpars matrix.
